# An Index to the Character of Medical Journals

**Published:** 1901-01

**Authors:** 


### AN INDEX TO THE CHARACTER OF MEDICAL JOURNALS.

THE *Journal* is once more in receipt of the generous annual advertising proposition of the publishers of the *Scientific American*. In exchange for advertising space during the year 1901 to the value of $45 they offer freely to send during the same period an exchange copy of their publication, whose subscription price is $3 a year. This generosity is appalling. It is however of interest to note that many "medical" journals accept this proposition with avidity. Subscribers and advertisers may readily find in the attidude of medical papers toward this proposition a fairly accurate index of the real value set by their publishers on space in the journals in whose columns this advertisement will appear. To this extent Messrs. Munn & Company are unconsciously doing the medical profession a service.

The above is taken from the December issue of the Cleveland *Medical Journal* and is inserted as an amusing instance of the glittering conceit and bumptious self-importance of this young and aspiring publication.

"The vain gods sigh for the cross and pain,
And the reed that grew grows never again
As a reed with the reeds in the river."

It is a brave journal that would hazard the frowning displeasure of this young giant of the West. It would mean swift and total annihilation for the offender.

Yet, the discovery that the presence of the *Scientific American* advertisement in a medical journal furnishes an index of its standing appears to contain more ingenuity than sense, and is only paralleled by the implied information that the small niche occupied by the cut in question is worth $45 in the Cleveland *Medical Journal*, of which there is nothing glaringly affirmatory in the side of the journal presented to the public. While confessing failure to see the force of the Cleveland *Journal's* discovery, we would fain express our conviction that this journal is not by unanimous consent the censor of medical journal morals or measures, and its self-assumed dictatorship is not only offensive, but reacts upon itself with all the equivocalities of self-praise. Were it not for this pharisaic self-righteousness, the Cleveland *Medical Journal* would meet with our approbation. We will even go so far as to say that it is one of the most admirable of the cheaper class of medical publications and probably occupies an enviable position in local medical circles. Some of its editorials are quite pleasing, even clever, but at times it is tiresome.

